# Supplementary material for: A robust fuzzy logic-based model for predicting the critical total drawdown in sand production in oil and gas wells
Source: PLoS One. 2021 Apr 26;16(4):e0250466. doi: 10.1371/journal.pone.0250466 (PMC8075206; doi:10.1371/journal.pone.0250466)
Supplement: S1 Appendix — (DOCX) [file pone.0250466.s001.docx]

**S1 Appendix**

**Coefficient of determination (R^2^)**

The following equation can determine the **R^2^**:

| $R^{2}=(\frac{n\sum xy-(\sum x)(\sum y)}{\sqrt{n(\sum x^{2})-(\sum x)^{2}}\sqrt{n(\sum y^{2})-(\sum y)^{2}}})^{2}$ | (1) |
| --- | --- |

**Relative deviation error**

The relative deviation error ($E_{i}$) of the predicted CTD value from the measured value is given as follows:

| $E_{i}=\frac{predictedCTD-measuredCTD}{measuredCTD}$ | (2) |
| --- | --- |

*i* = 1, 2, 3,..., n.

**APRE**

APRE calculates the sum of the relative deviation in terms of percentage. APRE can be determined from Equation 3:

| $E_{r}=(\frac{1}{n})\sum_{i=1}^{n} E_{i}$ | (3) |
| --- | --- |

**AAPRE**

AAPRE can be calculated from Equation 4:

| $E_{a}=(\frac{1}{n})\sum_{i=1}^{n} \left\vert E_{i}\right\vert$ | (4) |
| --- | --- |

**Correlation coefficient (R)**

It can be used as an indicator to compare the measured CTD value with the predicted CTD value. It can be determined from:

| $R=\sqrt{1-\frac{\sum_{i=1}^{n} \left[measuredCTD-predictedCTD\right]}{\sum_{i=1}^{n} \left[measuredCTD-\overline{▷}CTD\right]}}$  where: $\overline{▷}CTD=\frac{1}{n}\sum_{i=1}^{N} \left[measuredCTD\right]_{i}$  | (5) |
| --- | --- |

**Standard deviation (SD)**

The SD can be defined as the measure of dispersion. The lower the value of the SD, the higher the accuracy of the model. It can be obtained by:

| $SD=\sqrt{\sum_{i=1}^{n} \frac{\left[\overline{x}_{errors}-x_{ierrors}\right]^{2}}{n-1}}$ | (6) |
| --- | --- |

**RMSE**

The equation of RMSE can be calculated as follows:

| $RMSE=\sqrt{\left[\frac{1}{n}\sum_{i=1}^{n} E_{i}^{2}\right]}$ | (7) |
| --- | --- |
