# Supplementary material for: A robust fuzzy logic-based model for predicting the critical total drawdown in sand production in oil and gas wells
Source: PLoS One. 2021 Apr 26;16(4):e0250466. doi: 10.1371/journal.pone.0250466 (PMC8075206; doi:10.1371/journal.pone.0250466)
Supplement: Attachment — Submitted filename: Response to Reviewers.docx [file pone.0250466.s002.docx]

**The novelty of the study**

Sand production from oil and gas reservoirs causes many problems that negatively impact petroleum facilities integrity and raise environmental and safety concerns. Therefore, early prediction of sand onset from reservoirs is essential to prepare the necessary measurements. This manuscript introduces a developed Fuzzy logic (FL) model for predicting sand production, which would minimise sand production in oil and gas reservoirs. The FL model has the best accuracy (0.9947 correlation coefficient and <10% AAPRE) in predicting the CTD and superior to other models (>20% AAPRE) in the literature. Also, the trend analysis showed that all the input parameters of the developed FL model could follow the correct trends, indicating the FL model’s reliability.

**Reviewer #1:** This paper presented a robust fuzzy logic-based model to predict the CTD of sand production in oil and gas wells. The research is interesting and rather important. However, there exist a number of issues throughout the manuscript, especially the especially literature review, the proposed methodology, etc., that need to be addressed or clarified before it can be accepted for publication at the journal.

The main issues are as follows.

| Comments reviewer | Answers |
| --- | --- |
| 1) In Methodology Section, the fuzzy control model shall be specifically designed for the CTD prediction instead of the general architecture of fuzzy inference system (Fig.1). The value of the parameters in the model shall be defined clearly, for example, the membership function, the fuzzy rules, the ways of fuzzification and defuzzification, etc. Besides, the improvement and advantages of the proposed model shall be fully demonstrated to highlight the contribution of the manuscript. | Done, you can see Fig. 1, Table 2 and the text highlighted in yellow colour. |
| 2) Line 49, Artificial Intelligence (AI)? I did not see any AI description in the manuscript. | Done. |
| 3) Line 58 - Line 106 , three techniques for predicting sand production are discussed. Are these techniques also used for predicting the CTD? Line 102 - 118 discussed the models to predict the CTD. The relationship of these two parts shall be explained. | The three techniques are used to predict sand production. The CTD is one of these methods (empirical method). You can see the text highlighted in yellow colour. |
| 4)Line 83, 84, etc, an FL approach? Or a FL approach. | An FL, done. |
| 5)Line 117, “the applicability of these models has significant limitations and their accuracy is questionable.” needs detailed explanation. | Done. You can see the text highlighted in yellow colour. |
| 6) The literature review is missing. The current study of CTD predicting and the FL method shall be provided. | Done. It is highlighted in yellow colour. |
| 7) Line 181, “the sensitivity analysis or trend analysis is essential for this study” is confusing. The title of this section is ‘sensitivity analysis’, however the result analysis is based on the trend analysis. | Done. |
